# DCAI: a dual cross-attention integration framework for benign-malignant classification of pulmonary nodules

**DOI:** 10.3389/fmed.2025.1636008

**Published:** 2025-07-21

**Authors:** Shuling Wang, Suixue Wang, Rongdao Sun

**Affiliations:** ^1^Department of Neurology, Haikou Affiliated Hospital of Central South University Xiangya School of Medicine, Haikou, China; ^2^School of Computer Science and Technology, Hainan University, Haikou, China

**Keywords:** pulmonary nodule, benign-malignant classification, artificial intelligence, multimodal, cross-attention, transformer

## Abstract

Lung cancer remains a leading cause of cancer-related mortality worldwide, and accurate early identification of malignant pulmonary nodules is critical for improving patient outcomes. Although artificial intelligence (AI) technology has shown promise in pulmonary nodule benign-malignant classification, existing methods struggle with modality heterogeneity and limited exploitation of complementary information across modalities. To address the above issues, we propose a novel multimodal framework, the Dual Cross-Attention Integration framework (DCAI), for benign-malignant classification of pulmonary nodules. Specifically, we first convert 3D nodules into multiple 2D images and obtain nodule features annotated by clinical experts. These features are encoded using Transformer models, and then a dual cross-attention module is proposed to dynamically align and interact with the complementary information between the different modalities. The fused representations from both modalities are then concatenated for benign-malignant prediction. We evaluate our proposed method on the LIDC-IDRI dataset, and experimental results demonstrate that DCAI outperforms several existing multimodal methods, highlighting the effectiveness of our approach in improving the accuracy of pulmonary nodule benign-malignant classification.

## 1 Introduction

Lung cancer remains the leading cause of cancer-related mortality worldwide, with its five-year survival rate strongly dependent on early diagnosis. According to the World Health Organization (WHO), lung cancer accounted for ~2.2 million new cases and 1.8 million deaths worldwide in 2020, accounting for 18% of all cancer deaths (1). Pulmonary nodules are critical radiographic indicators of early-stage lung cancer, and accurate differentiation between benign and malignant nodules is essential for clinical decision-making and patient prognosis. Low-dose computed tomography (LDCT), the primary screening modality, has significantly improved detection rates, but it also results in a high false-positive rate of up to 95% (2), leading to unnecessary invasive procedures (e.g., biopsy) and increased strain on healthcare resources. Conventional diagnosis relies heavily on radiologists' subjective assessment of nodule morphological features (e.g., spiculation, lobulation), which is limited by inter-observer variability (Cohen's *k* = 0.45–0.67) (3) and reduced sensitivity for small nodules (< 6 mm) (4). Although adjunctive techniques such as PET-CT and liquid biopsy can provide additional diagnostic information, their clinical utility is constrained by high costs, radiation exposure, and risks associated with invasive procedures (5).

Hence, the development of efficient and noninvasive methods for predicting the malignancy of pulmonary nodules has become a major research focus. Radiomics, which quantitatively analyzes features such as texture, shape, and heterogeneity of nodules, has significantly enhanced diagnostic objectivity. For instance, the American College of Radiology (ACR) Lung-RADS classification system standardizes the evaluation process, increasing early lung cancer diagnostic specificity to 85%. However, its positive predictive value for category 3–4 nodules remains below 35% (5). Recent advances in artificial intelligence (AI) offer new opportunities for automated benign-malignant classification of pulmonary nodules with multimodal data, such as CT images and structured features annotated by the clinician. Deep learning models (6–8) can extract high-level features from multimodal data beyond human visual perception, with studies showing that AI-assisted systems can achieve classification accuracies exceeding 90% for small nodules, thereby reducing overdiagnosis and improving resource allocation (9).

Despite progress in existing methods, multimodal fusion still faces three major challenges: (1) The modality heterogeneity between CT images (high-dimensional spatial data) and clinical features (low-dimensional structured data) complicates feature alignment. (2) Traditional fusion strategies, such as concatenation or weighted averaging, struggle to dynamically adjust for redundant or conflicting information. To address the above issues, we propose a dual cross-attention integration framework, named DCAI, to classify the benign-malignant of pulmonary nodules. Specifically, 3D nodule CT scans are first converted into multiple 2D slices, and expert-annotated clinical features are interpolated. Both modalities are encoded using Transformer models, and a dual cross-attention module is proposed to capture complementary information between them. The resulting fused representations are concatenated for benign-malignant prediction. Experimental results demonstrate the superior performance of DCAI over existing multimodal methods, highlighting its effectiveness in improving pulmonary nodule benign-malignant classification accuracy.

In summary, our contributions are as follows:

- We incorporate transformer models to deeply encode clinical structured data and CT images separately, enabling the learning of high-level features from both modalities.
- We propose a dual cross-attention module that dynamically regulate the flow of complementary information between imaging and clinical structured features, effectively addressing modality heterogeneity and alignment challenges.

## 2 Related work

In recent years, research on pulmonary nodule malignancy classification has evolved along two major technical pathways: unimodal and multimodal approaches. In unimodal methods, most studies focus on developing deep learning models based solely on CT imaging. For instance, Li et al. (10) a deep convolutional neural network for nodule classification that leverages automatic feature learning and exhibits strong generalization performance. Donga et al. (11) propose a machine learning-based framework using a modified gradient boosting method for classifying pulmonary nodules, which integrates CT image preprocessing, random walker segmentation, and feature extraction. Wang et al. (12) proposed a decision tree model based on nodule size and density thresholds for preliminary risk stratification in the Chinese population, achieving an AUC of 0.899 in the first phase of their C-Lung-RADS system. However, unimodal approaches face challenges in handling the complexity and variability of pulmonary nodules, including high intra- and inter-patient variability in shape, size, and texture. Small or early-stage nodules may lack distinct features in CT scans, leading to reduced sensitivity and accuracy in malignancy prediction.

Multimodal approaches further enhance performance by integrating imaging data with clinical information. A notable example is the work by Yao et al. (13), who introduced a machine learning framework combining dynamic PET/CT metabolic and hemodynamic features, such as time-activity curve decomposition (TAC), to improve diagnostic specificity. Recent innovations also explore cross-modal fusion, such as the RFSC network developed by Wang et al., which aligns low-dose CT and MRI images through unsupervised registration, achieving 89.9% classification accuracy while reducing radiation exposure (14). Yuan et al. (15) propose a multi-modal fusion multi-branch classification network, which integrates structured radiological features and 3D CT patch data using an effective attention mechanism to classify pulmonary nodules as benign or malignant. These multimodal frameworks address the limitations of unimodal methods by leveraging complementary data sources, thereby reducing false positives and optimizing resource allocation in clinical practice. Sun et al. (16) propose the Nodule-CLIP model, which leverages comparative learning to explore the relationship between CT images and lung nodule attributes, enhancing the model's ability to distinguish between benign and malignant nodules. Tang et al. (17) construct two models (SUDFNN and SUDFX) that integrate 3D CNN-extracted image features with radiologist-annotated structured features using softmax and XGBoost classifiers, respectively. Liu et al. (18) propose a multimodal deep learning network integrating ResNet imaging features, Word2Vec semantic data, and self-attention mechanisms, achieving high accuracy in differentiating benign/malignant pulmonary nodules.

## 3 Method

### 3.1 Data preprocessing

The experimental data were derived from two publicly available datasets: the Lung Image Database Consortium and Image Database Resource Initiative (LIDC-IDRI) (3) and the Lung Nodule Analysis 2016 (LUNA16) (19). The LIDC-IDRI dataset comprises 1,018 thoracic CT scans with XML annotations generated through a two-phase review protocol involving four board-certified radiologists. Annotated nodule characteristics include malignancy, subtlety, internal structure, calcification, sphericity, margin, lobulation, speculation, texture, and diameter (the latter provided by LUNA16).

Following previous work (17), we consider the criteria required nodules ≥3 mm in diameter with consensus annotations from at least three radiologists. The malignancy score (ranging 1–5, higher values indicating increased malignancy likelihood) served as the classification target. For each nodule, multi-reader malignancy ratings are averaged and rounded to the nearest integer. Nodules with final scores of 1–2 are categorized as benign (*n* = 354), while those scoring 4–5 are classified as malignant (*n* = 330). Intermediate scores (3) are excluded to ensure diagnostic certainty. Missing annotations in other structured characteristics are addressed through radiologist-wise imputation. When only three radiologists provided annotations, the fourth radiologist's entry is populated using the rounded mean of existing annotations. CT image preprocessing involved isotropic resampling to 1 × 1 × 1*mm*^3^/*voxel* resolution, followed by extraction of 32 × 32 × 32 voxel cubes centered on nodule coordinates. This yielded standardized 3D nodule volumes as unstructured imaging inputs. As a result, we obtain 684 samples, each comprising nine structured radiographic attributes, one 3D nodule volume, and a binary benign/malignant label.

### 3.2 Dual cross-attention integration framework

**Figure 1:**
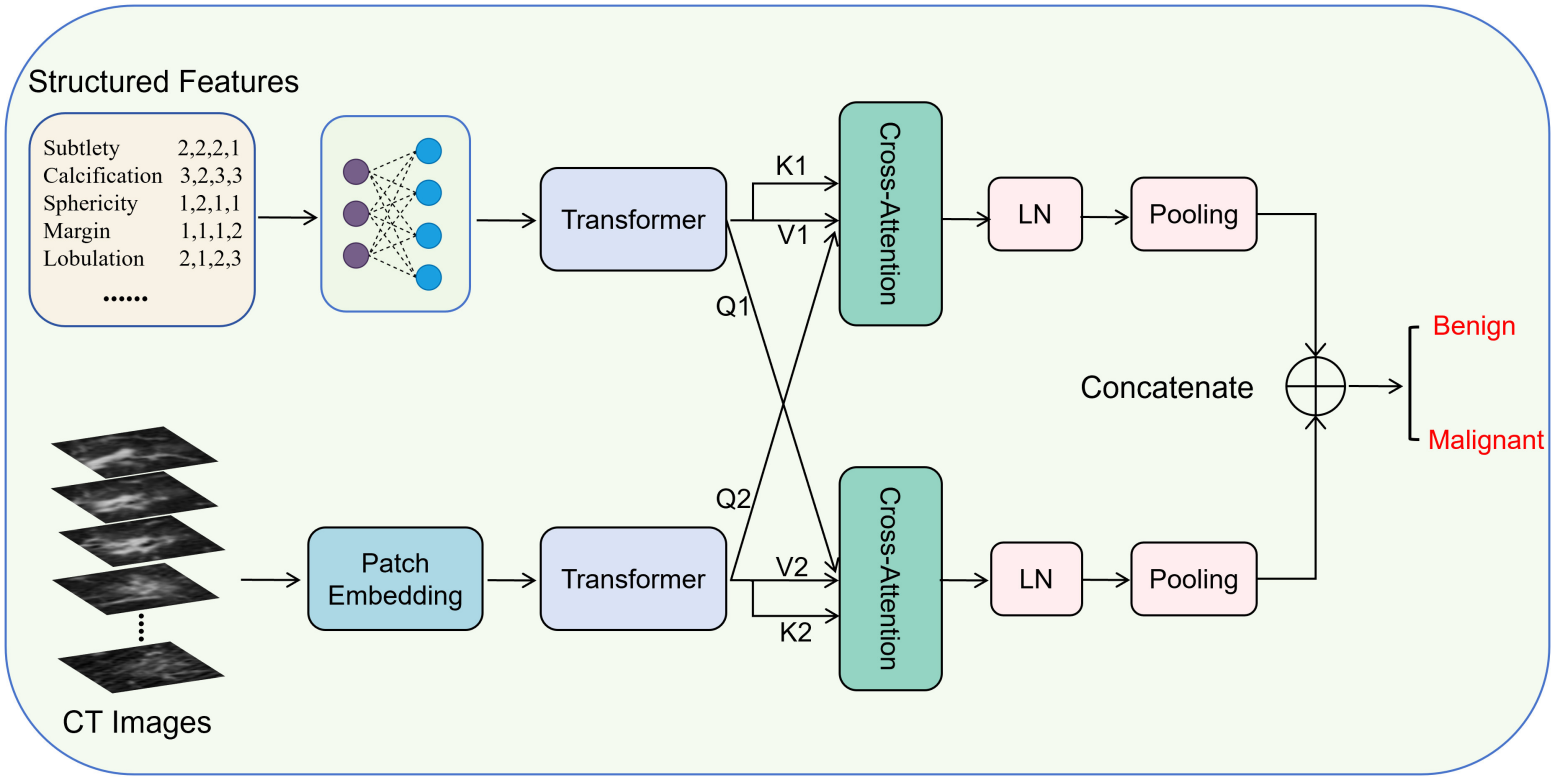
The overall framework of dual cross-attention integration (DCAI).

The overall framework of DCAI is illustrated in Figure 1. Given a nodule case *x*_*i*_ from the dataset *x* = {*x*_1_, *x*_2_, …, *x*_*N*_}, containing a 3D nodule volume (CT image) $c_{i}\in {\mathbb{R}}^{32\times 32\times 32}$ and nine structured features $s_{i}\in {\mathbb{R}}^{9\times 4}$, we encode them separately into representations using two types of encoders, which are then aligned with two dual cross-attention modules and fused with concatenation.

Specifically, we employ a linear network built on two fully connected layers to map the original structured features to a new semantic space, and then learn the relationships within the structured features using a Transformer module. They are written as:


(1) $$\begin{array}{l} S_{emb}=\text{ReLU}\left({\text{W}}_{2}\left({\text{W}}_{1}\text{S}+{\text{b}}_{1}\right)+{\text{b}}_{2}\right) \end{array}$$

where ${\text{W}}_{1}\in {\mathbb{R}}^{4\times 32}$, ${\text{W}}_{2}\in {\mathbb{R}}^{32\times 256}$, ${\text{b}}_{1}\in {\mathbb{R}}^{1\times 32}$, and ${\text{b}}_{2}\in {\mathbb{R}}^{1\times 256}$ are learnable weights. ReLU is the activation function.


(2) $$\begin{array}{l} S_{enc}=\text{Transformer-S}\left(S_{emb}\right) \end{array}$$

where Transformer-S() is the Transformer encoder module, which consists of 6 Transformer blocks.

Meanwhile, 3D nodule volume (CT image) is first divided into 32 32 × 32 images, and then patch embedded with a lightweight CNN, followed by encoding with a Transformer (20, 21), which are written as:


(3) $$\begin{array}{l} C_{emb}=\text{LightweightCNN}\left(C\right) \end{array}$$

(4) $$\begin{array}{l} C_{enc}=\text{Transformer-C}\left(C_{emb}\right) \end{array}$$

where LightweightCNN denotes the lightweight CNN with a kernel size of 32 × 32 and 256 channels.

After that, the encoded representations of structured features and CT image are aligned and dynamically interact with two dual cross-attention modules (22, 23), which are written as:


(5) $$\begin{array}{l} \begin{array}{c} S_{CA}=\text{CrossAttn}\left({\text{W}}_{q,s}C_{enc},{\text{W}}_{k,s}{\text{S}}_{enc},{\text{W}}_{v,s}S_{enc}\right)\\ \;=\text{Softmax}\left(\frac{{\text{W}}_{q,s}C_{enc}S_{enc}^{T}{\text{W}}_{k,s}^{T}}{\sqrt{d}}\right){\text{W}}_{v,s}S_{enc} \end{array} \end{array}$$

(6) $$\begin{array}{l} \begin{array}{c} C_{CA}=\text{CrossAttn}\left({\text{W}}_{q,c}{\text{S}}_{enc},{\text{W}}_{k,c}{\text{C}}_{enc},{\text{W}}_{v,c}{\text{C}}_{enc}\right)\\ \;=\text{Softmax}\left(\frac{{\text{W}}_{q,c}{\text{S}}_{enc}{\text{C}}_{enc}^{T}{\text{W}}_{k,c}^{T}}{\sqrt{d}}\right){\text{W}}_{v,c}{\text{C}}_{enc} \end{array} \end{array}$$

where W_*q, s*_, W_*k, s*_, W_*v, s*_, W_*q, c*_, W_*k, c*_, and W_*v, c*_ are trainable weight matrices multiplied by the corresponding queries, keys, and values.

Then, the *S*_*CA*_ and *C*_*CA*_ are layer normalizated and average pooled, respectively. Next, the two unimodal representations are concatenated as the multimodal representations:


(7) $$\begin{array}{l} S_{final}=\text{AvgPool}\left(\text{LayerNorm}\left(S_{CA}\right)\right) \end{array}$$

(8) $$\begin{array}{l} C_{final}=\text{AvgPool}\left(\text{LayerNorm}\left(C_{CA}\right)\right) \end{array}$$

(9) $$\begin{array}{l} V=\left[S_{final}⊕C_{final}\right] \end{array}$$

Finally, the multimodal representations V are used to predict the probabilities of nodule samples. Specifically, the multimodal representations V are first fed into a linear network, followed by the application of a cross-entropy loss function to compute the final loss. The overall process is formulated as follows:


(10) $$\begin{array}{l} L=\text{CE}\left(\text{Linear}\left(V\right),y^{true}\right) \end{array}$$

where *y*^*true*^ is the ground-truth labeling and CE(·) denotes the cross-entropy loss function.

## 4 Experiments and results analysis

### 4.1 Experiment setup

The experiments are executed on a Linux-based system equipped with three NVIDIA A100 GPUs and the PyTorch framework. The DCAI model is trained for 30 epochs with a batch size of 100, using a learning rate of 0.0005 and a weight decay of 0.001 to regularize optimization. The Transformer architecture comprised six stacked encoder blocks, ensuring sufficient depth for feature abstraction.

We conduct all experiments using a rigorous five-fold cross-validation to ensure robust performance evaluation. The dataset is randomly partitioned into five distinct subsets, with each fold serving as the test set once, while the remaining four folds are utilized for model training. The final performance metrics are averaged across all five iterations to mitigate bias and enhance statistical reliability.

### 4.2 Evaluation metrics

We evaluate the performance of pulmonary nodule malignancy classification (malignant as positive, benign as negative) by various metrics, such as accuracy, precision, sensitivity, specificity, and F1-Score. They are calculated based on a confusion matrix:


(11) $$\begin{array}{l} \text{Confusion-Matrix}=\left[\begin{array}{cc} \text{TN} & \text{FP}\\ \text{FN} & \text{TP} \end{array}\right] \end{array}$$

where TN (true negative) and TP (true positive) denote correct predictions for benign and malignant cases, respectively; FP (False Positive) and FN (False Negative) represent misclassified benign and malignant cases.

The metrics of accuracy, precision, sensitivity, specificity, and F1-Score are written as:


(12) $$\begin{array}{l} \text{Accuracy}=\frac{\text{TP}+\text{TN}}{\text{TP}+\text{TN}+\text{FP}+\text{FN}} \end{array}$$

(13) $$\begin{array}{l} \text{Precision}=\frac{\text{TP}}{\text{TP}+\text{FP}} \end{array}$$

(14) $$\begin{array}{l} \text{Sensitivity}=\frac{\text{TP}}{\text{TP}+\text{FN}} \end{array}$$

(15) $$\begin{array}{l} \text{Specificity}=\frac{\text{TN}}{\text{TN}+\text{FP}} \end{array}$$

(16) $$\begin{array}{l} \text{F1-Score}=2\times \frac{\text{Precision}\times \text{Recall}}{\text{Precision}+\text{Recall}} \end{array}$$

### 4.3 Comparison with existing multimodal methods

To validate the effectiveness of our method, we compare DCAI with three multimodal fusion approaches: Nodule-CLIP (16), Self-attention-based (18), and SUDFX32 (17). As shown in Table 1, DCAI achieves superior performance across all metrics, attaining an accuracy of 0.964, precision of 0.955, sensitivity of 0.970, specificity of 0.958, and F1-score of 0.962. Notably, DCAI outperforms SUDFX32, the previous best method, by 2.2% in accuracy and 2.2% in F1-score, demonstrating its robust capability in integrating multimodal features. The significant improvements in sensitivity (1.5% higher than SUDFX32) and specificity (2.8% higher) further highlight its balanced diagnostic reliability. These results validate the efficacy of our cross-modal alignment strategy, which effectively reduce inter-modal discrepancies while preserving discriminative features.

**Table 1:** Performance comparison of DCAI and existing models on the LIDC-IDRI dataset.

**Methods**	**Accuracy**	**Precision**	**Sensitivity**	**Specificity**	**F1-Score**
Nodule-CLIP (16)	0.934	0.925	0.939	0.930	0.932
Self-attention-based (18)	0.920	0.910	0.924	0.915	0.917
SUDFX32 (17)	0.942	0.926	0.955	0.930	0.940
DCAI (Ours)	**0.964**	**0.955**	**0.970**	**0.958**	**0.962**

### 4.4 Ablation study

To investigate the performance of different modal input data and individual submodules, we conduct ablation experiments on Unimodal vs. Multimodal configurations and Key Components, respectively.

#### 4.4.1 Unimodal and multimodal

Table 2 reveals distinct strengths of unimodal inputs: structured features excel in precision (0.967), indicating robust identification of benign cases, while CT images achieve superior sensitivity (0.985), effectively detecting malignant nodules. However, unimodal models exhibit limitations–structured features show lower sensitivity (0.879), and CT images underperform in specificity (0.901). Multimodal fusion balances these metrics, achieving optimal accuracy (0.964) and F1-score (0.962). This synergy highlights how combining clinical metadata with imaging data mitigates modality-specific biases, enhancing holistic diagnostic reliability for both benign and malignant cases.

**Table 2:** Ablation study of modality.

**Modality**	**Accuracy**	**Precision**	**Sensitivity**	**Specificity**	**F1-Score**
Structured features	0.927	**0.967**	0.879	**0.972**	0.921
CT images	0.942	0.903	**0.985**	0.901	0.942
Multimodal	**0.964**	0.955	0.970	0.958	**0.962**

#### 4.4.2 Key components

Ablating key components (Table 3) demonstrates their critical roles. Removing the Transformer reduces specificity (0.901 vs. 0.958), suggesting its necessity for modeling global context to minimize false positives (benign misclassified as malignant). Disabling cross-attention causes significant drops in sensitivity (0.909 vs. 0.970) and precision (0.882 vs. 0.955), emphasizing its role in aligning multimodal features for accurate malignant detection. The complete DCAI model architecture achieves balanced performance, proving that both components are crucial for dynamic cross-modal interaction and discriminative feature preservation.

**Table 3:** Ablation study of key components.

**Methods**	**Accuracy**	**Precision**	**Sensitivity**	**Specificity**	**F1-Score**
Without transformer	0.927	0.900	0.955	0.901	0.926
Without cross-attention	0.898	0.882	0.909	0.887	0.896
DCAI (complete)	**0.964**	**0.955**	**0.970**	**0.958**	**0.962**

## 5 Conclusion

In this paper, we propose DCAI, a dual cross-attention integration framework for benign-malignant classification of pulmonary nodules. We design DCAI to address modality heterogeneity and feature alignment challenges in multimodal fusion. Leveraging Transformer-based encoders for clinical structured features and CT images, DCAI captures high-level semantic representations while dynamically aligning complementary information through the dual cross-attention module. Evaluated on the LIDC-IDRI dataset, DCAI achieves superior performance, outperforming existing methods significantly. Ablation studies confirm the necessity of both cross-attention and Transformer components. The experimental results indicate that our framework provides a robust, noninvasive solution to enhance early lung cancer diagnosis and reduce unnecessary interventions, demonstrating clinical potential for reliable malignancy characterization.

## Data Availability

The original contributions presented in the study are included in the article/supplementary material, further inquiries can be directed to the corresponding author.
